# 6-Nitro-1,3-benzothia­zole-2(3*H*)-thione

**DOI:** 10.1107/S1600536812049719

**Published:** 2013-01-04

**Authors:** Qi-Ming Qiu, Yang-Zhe Cui, Yin-Hua Zhao, Qiong-Hua Jin, Cun-Lin Zhang

**Affiliations:** a Department of Chemistry, Capital Normal University, Beijing 100048, People’s Republic of China; b Key Laboratory of Terahertz Optoelectronics, Ministry of Education, Department of Physics, Capital Normal University, Beijing 100048, People’s Republic of China

## Abstract

In the title mol­ecule, C_7_H_4_N_2_O_2_S_2_, the nitro group is twisted by 5.5 (1)° from the plane of the attached benzene ring. In the crystal, N—H⋯S hydrogen bonds link pairs of mol­ecules into inversion dimers, which are linked by weak C—H⋯O inter­actions into sheets parallel to (101). The crystal packing exhibits short inter­molecular S⋯O contacts of 3.054 (4) Å and π–π inter­actions of 3.588 (5) Å between the centroids of the five- and six-membered rings of neighbouring mol­ecules.

## Related literature

For coordination compounds based on 2-mercapto-6-nitro­benzothia­zole ligands, see: Ma *et al.* (2003*a* ▶,*b* ▶, 2004 ▶). For the structure of the related compound 2-mercapto-benzothia­zole, see: Chesick & Donohue (1971 ▶).
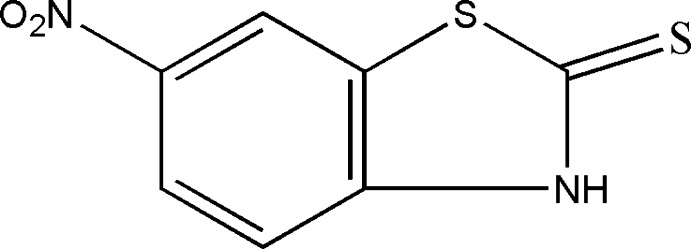


## Experimental

### 

#### Crystal data

C_7_H_4_N_2_O_2_S_2_
*M* *_r_* = 212.24
Monoclinic, 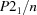

*a* = 3.8645 (2) Å
*b* = 26.345 (2) Å
*c* = 7.8961 (4) Å
β = 92.509 (1)°
*V* = 803.14 (9) Å^3^
*Z* = 4
Mo *K*α radiation
μ = 0.62 mm^−1^
*T* = 298 K
0.40 × 0.35 × 0.27 mm

#### Data collection

Bruker SMART CCD area-detector diffractometer
Absorption correction: multi-scan (*SADABS*; Bruker, 2007 ▶) *T*_min_ = 0.789, *T*_max_ = 0.850
4092 measured reflections
1425 independent reflections
1104 reflections with *I* > 2σ(*I*)
*R*_int_ = 0.109

#### Refinement

*R*[*F* ^2^ > 2σ(*F* ^2^)] = 0.063
*wR*(*F* ^2^) = 0.165
*S* = 1.04
1425 reflections
118 parameters
H-atom parameters constrained
Δρ_max_ = 0.45 e Å^−3^
Δρ_min_ = −0.42 e Å^−3^

### 

Data collection: *SMART* (Bruker, 2007 ▶); cell refinement: *SAINT-Plus* (Bruker, 2007 ▶); data reduction: *SAINT-Plus*; program(s) used to solve structure: *SHELXS97* (Sheldrick, 2008 ▶); program(s) used to refine structure: *SHELXL97* (Sheldrick, 2008 ▶); molecular graphics: *SHELXTL* (Sheldrick, 2008 ▶); software used to prepare material for publication: *SHELXTL*.

## Supplementary Material

Crystal structure: contains datablock(s) global, I. DOI: 10.1107/S1600536812049719/cv5367sup1.cif

Structure factors: contains datablock(s) I. DOI: 10.1107/S1600536812049719/cv5367Isup2.hkl

Supplementary material file. DOI: 10.1107/S1600536812049719/cv5367Isup3.cml

Additional supplementary materials:  crystallographic information; 3D view; checkCIF report

## Figures and Tables

**Table 1:** Hydrogen-bond geometry (Å, °)

*D*—H⋯*A*	*D*—H	H⋯*A*	*D*⋯*A*	*D*—H⋯*A*
N1—H1⋯S2^i^	0.86	2.45	3.271 (3)	160
C4—H4⋯O1^ii^	0.93	2.60	3.285 (6)	131

Symmetry codes: (i) 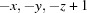; (ii) 
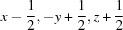
.

**Fig. 1.**
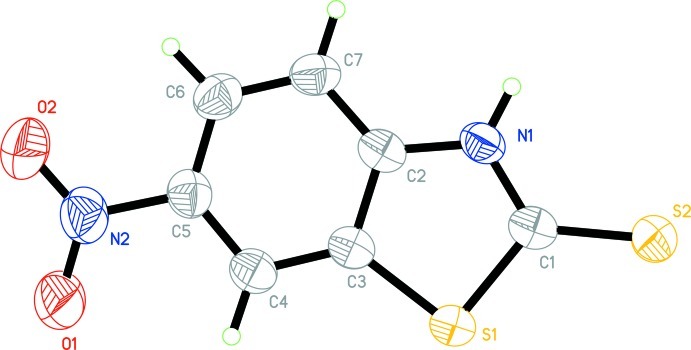
The molecular structure of (I) showing the atomic numbering scheme and displacement ellipsoids drawn at the 50% probability level.

**Fig. 2.**
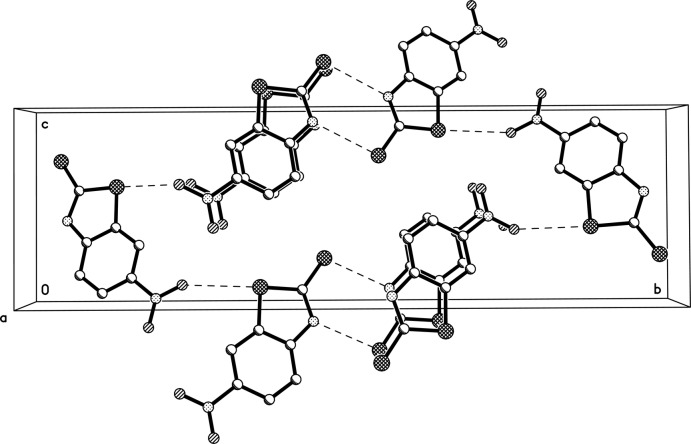
A portion of the crystal packing viewed approximately along the a axis. Dashed lines indicate short N···S and O···S contacts. H atoms omitted for clarity.

## supplementary crystallographic information

### Comment

Metal-organic supramolecular compounds have received much attention due to their structural diversities and potential applications as new materials. The ligand 2-mercapto-6-nitrobenzothiazole (MNBT) is excellent in building supramolecular structures. However, to our best knowledge, only a few Ag(I)-MNBT (MNBT = 2-mercapto-6-nitrobenzothiazole) framework structures have been reported (Ma *et al.*, 2003*a*,*b*; 2004). The title compound, (I), was unexpectedly obtained when we tried to synthesize Ag(I)-MNBT complexes containing bis(diphenylphosphino)methane.

In (I) (Fig.1), the nitro group is twisted at 5.5 (1)° from the plane of the attached benzene ring. Intermolecular N—H···S hydrogen bonds (Table 1) link two molecules into centrosymmetric dimer, and weak C—H···O interactions (Table 1) link further these dimers into sheets parallel to (101). The hydrogen bond N—H···S is similar to that reported for 2-mercapto-benzothiazole (Chesick & Donohue, 1971). The crystal packing (Fig. 2) exhibits short intermolecular S···O contacts of 3.054 (4) Å and π–π interactions proved by short distance of 3.588 (5) Å between the centroids of the five- and six-membered rings from the neighbouring molecules.

### Experimental

A mixture of AgCl (0.2 mmol) and bis(diphenylphosphino)methane (0.2 mmol) in MeOH and CH_2_Cl_2_ (10 mL, *v*/*v* = 1:1) was stirred for 3 h. The insoluble residues were removed by filtration. The filtrate was then evaporated slowly at room temperature for a week to yield colourless crystalline product.

The title compound was prepared by dissolving 0.0587 g colourless product mentioned above in MeOH and CH_2_Cl_2_ (10 mL, *v*/*v* = 3:7), adding 2-mercapto-6-nitrobenzothiazole (0.2 mmol) into the solution, stirring for 4 h. Subsequent slow evaporation of the yellow filtrate resulted in the formation of yellow crystals.

### Refinement

All H atoms were geometrically positioned [C—H 0.93 Å; N—H 0.86 Å], and included in the final refinement in the riding model approximation, with *U*_iso_(H) = 1.2 *U*_eq_ of the parent atom.

### Crystal data

**Table:**

C_7_H_4_N_2_O_2_S_2_	*F*(000) = 432
*M**_r_* = 212.24	*D*_x_ = 1.755 Mg m^−^^3^
Monoclinic, *P*2_1_/*n*	Mo *K*α radiation, λ = 0.71073 Å
Hall symbol: -P 2yn	Cell parameters from 1148 reflections
*a* = 3.8645 (2) Å	θ = 2.7–23.7°
*b* = 26.345 (2) Å	µ = 0.62 mm^−^^1^
*c* = 7.8961 (4) Å	*T* = 298 K
β = 92.509 (1)°	Block, colourless
*V* = 803.14 (9) Å^3^	0.40 × 0.35 × 0.27 mm
*Z* = 4	

### Data collection

**Table:**

Bruker SMART CCD area-detector diffractometer	1425 independent reflections
Radiation source: fine-focus sealed tube	1104 reflections with *I* > 2σ(*I*)
Graphite monochromator	*R*_int_ = 0.109
phi and ω scans	θ_max_ = 25.0°, θ_min_ = 2.7°
Absorption correction: multi-scan (*SADABS*; Bruker, 2007)	*h* = −4→4
*T*_min_ = 0.789, *T*_max_ = 0.850	*k* = −30→31
4092 measured reflections	*l* = −9→6

### Refinement

**Table:**

Refinement on *F*^2^	Primary atom site location: structure-invariant direct methods
Least-squares matrix: full	Secondary atom site location: difference Fourier map
*R*[*F*^2^ > 2σ(*F*^2^)] = 0.063	Hydrogen site location: inferred from neighbouring sites
*wR*(*F*^2^) = 0.165	H-atom parameters constrained
*S* = 1.04	*w* = 1/[σ^2^(*F*_o_^2^) + (0.0915*P*)^2^] where *P* = (*F*_o_^2^ + 2*F*_c_^2^)/3
1425 reflections	(Δ/σ)_max_ < 0.001
118 parameters	Δρ_max_ = 0.45 e Å^−^^3^
0 restraints	Δρ_min_ = −0.42 e Å^−^^3^

### Special details

**Table:**

Geometry. All e.s.d.'s (except the e.s.d. in the dihedral angle between two l.s. planes) are estimated using the full covariance matrix. The cell e.s.d.'s are taken into account individually in the estimation of e.s.d.'s in distances, angles and torsion angles; correlations between e.s.d.'s in cell parameters are only used when they are defined by crystal symmetry. An approximate (isotropic) treatment of cell e.s.d.'s is used for estimating e.s.d.'s involving l.s. planes.
Refinement. Refinement of *F*^2^ against ALL reflections. The weighted *R*-factor *wR* and goodness of fit *S* are based on *F*^2^, conventional *R*-factors *R* are based on *F*, with *F* set to zero for negative *F*^2^. The threshold expression of *F*^2^ > σ(*F*^2^) is used only for calculating *R*-factors(gt) *etc*. and is not relevant to the choice of reflections for refinement. *R*-factors based on *F*^2^ are statistically about twice as large as those based on *F*, and *R*- factors based on ALL data will be even larger.

### Fractional atomic coordinates and isotropic or equivalent isotropic displacement parameters (Å^2^)

**Table:**

	*x*	*y*	*z*	*U*_iso_*/*U*_eq_	
N1	0.2970 (8)	0.05823 (12)	0.4296 (4)	0.0383 (8)	
H1	0.2434	0.0278	0.3985	0.046*	
N2	0.8798 (11)	0.20738 (15)	0.0513 (6)	0.0559 (10)	
O1	0.9284 (13)	0.24753 (15)	0.1198 (5)	0.1031 (17)	
O2	0.9722 (12)	0.19831 (14)	−0.0880 (5)	0.0844 (13)	
S1	0.3759 (3)	0.13687 (4)	0.60511 (13)	0.0414 (4)	
S2	0.0806 (3)	0.04194 (4)	0.73917 (14)	0.0450 (4)	
C1	0.2447 (10)	0.07491 (14)	0.5857 (5)	0.0360 (9)	
C2	0.4399 (10)	0.09190 (14)	0.3216 (5)	0.0350 (9)	
C3	0.5018 (10)	0.13824 (14)	0.3978 (5)	0.0350 (9)	
C4	0.6477 (10)	0.17709 (15)	0.3110 (5)	0.0403 (10)	
H4	0.6936	0.2085	0.3610	0.048*	
C5	0.7219 (11)	0.16726 (15)	0.1476 (5)	0.0410 (10)	
C6	0.6620 (11)	0.12160 (16)	0.0691 (6)	0.0444 (10)	
H6	0.7212	0.1168	−0.0426	0.053*	
C7	0.5160 (11)	0.08351 (16)	0.1556 (5)	0.0429 (10)	
H7	0.4684	0.0524	0.1041	0.052*	

### Atomic displacement parameters (Å^2^)

**Table:**

	*U*^11^	*U*^22^	*U*^33^	*U*^12^	*U*^13^	*U*^23^
N1	0.043 (2)	0.0305 (17)	0.0407 (19)	−0.0046 (14)	−0.0031 (16)	−0.0022 (15)
N2	0.066 (3)	0.043 (2)	0.060 (3)	0.0034 (18)	0.013 (2)	0.010 (2)
O1	0.182 (5)	0.045 (2)	0.088 (3)	−0.028 (2)	0.066 (3)	−0.007 (2)
O2	0.129 (4)	0.067 (2)	0.060 (2)	−0.016 (2)	0.038 (2)	0.008 (2)
S1	0.0487 (7)	0.0354 (6)	0.0401 (6)	−0.0059 (4)	0.0029 (5)	−0.0051 (4)
S2	0.0497 (7)	0.0425 (6)	0.0427 (6)	−0.0076 (5)	0.0023 (5)	0.0004 (4)
C1	0.028 (2)	0.035 (2)	0.043 (2)	0.0026 (16)	−0.0063 (17)	−0.0030 (17)
C2	0.029 (2)	0.033 (2)	0.042 (2)	0.0016 (16)	−0.0089 (17)	−0.0010 (17)
C3	0.031 (2)	0.032 (2)	0.041 (2)	0.0034 (15)	−0.0022 (17)	−0.0017 (16)
C4	0.042 (2)	0.033 (2)	0.045 (2)	0.0029 (17)	0.0004 (19)	−0.0005 (17)
C5	0.040 (2)	0.037 (2)	0.047 (2)	0.0041 (17)	0.0051 (19)	0.0043 (19)
C6	0.050 (3)	0.047 (2)	0.035 (2)	0.005 (2)	0.0008 (19)	−0.0003 (19)
C7	0.049 (3)	0.041 (2)	0.038 (2)	0.0025 (18)	−0.0031 (19)	−0.0065 (19)

### Geometric parameters (Å, º)

**Table:**

N1—C1	1.332 (5)	C2—C7	1.373 (5)
N1—C2	1.364 (5)	C2—C3	1.378 (5)
N1—H1	0.8600	C3—C4	1.367 (5)
N2—O2	1.196 (5)	C4—C5	1.359 (5)
N2—O1	1.199 (5)	C4—H4	0.9300
N2—C5	1.452 (5)	C5—C6	1.368 (6)
S1—C1	1.714 (4)	C6—C7	1.351 (6)
S1—C3	1.728 (4)	C6—H6	0.9300
S2—C1	1.641 (4)	C7—H7	0.9300
			
C1—N1—C2	116.5 (3)	C4—C3—S1	129.0 (3)
C1—N1—H1	121.8	C2—C3—S1	110.2 (3)
C2—N1—H1	121.8	C5—C4—C3	116.3 (4)
O2—N2—O1	123.0 (4)	C5—C4—H4	121.9
O2—N2—C5	119.1 (4)	C3—C4—H4	121.9
O1—N2—C5	117.9 (4)	C4—C5—C6	124.0 (4)
C1—S1—C3	91.69 (18)	C4—C5—N2	118.0 (4)
N1—C1—S2	126.0 (3)	C6—C5—N2	118.0 (4)
N1—C1—S1	109.9 (3)	C7—C6—C5	119.4 (4)
S2—C1—S1	124.1 (2)	C7—C6—H6	120.3
N1—C2—C7	127.0 (4)	C5—C6—H6	120.3
N1—C2—C3	111.7 (3)	C6—C7—C2	118.3 (4)
C7—C2—C3	121.3 (4)	C6—C7—H7	120.8
C4—C3—C2	120.8 (4)	C2—C7—H7	120.8
			
C2—N1—C1—S2	−179.4 (3)	S1—C3—C4—C5	179.8 (3)
C2—N1—C1—S1	0.5 (4)	C3—C4—C5—C6	−0.6 (6)
C3—S1—C1—N1	−0.3 (3)	C3—C4—C5—N2	−179.5 (4)
C3—S1—C1—S2	179.6 (3)	O2—N2—C5—C4	174.2 (4)
C1—N1—C2—C7	−179.7 (4)	O1—N2—C5—C4	−1.9 (7)
C1—N1—C2—C3	−0.4 (5)	O2—N2—C5—C6	−4.8 (7)
N1—C2—C3—C4	179.4 (4)	O1—N2—C5—C6	179.2 (5)
C7—C2—C3—C4	−1.3 (6)	C4—C5—C6—C7	1.0 (7)
N1—C2—C3—S1	0.2 (4)	N2—C5—C6—C7	179.8 (4)
C7—C2—C3—S1	179.5 (3)	C5—C6—C7—C2	−1.4 (6)
C1—S1—C3—C4	−179.1 (4)	N1—C2—C7—C6	−179.2 (4)
C1—S1—C3—C2	0.1 (3)	C3—C2—C7—C6	1.6 (6)
C2—C3—C4—C5	0.7 (6)		

### Hydrogen-bond geometry (Å, º)

**Table:**

*D*—H···*A*	*D*—H	H···*A*	*D*···*A*	*D*—H···*A*
N1—H1···S2^i^	0.86	2.45	3.271 (3)	160
C4—H4···O1^ii^	0.93	2.60	3.285 (6)	131

Symmetry codes: (i) −*x*, −*y*, −*z*+1; (ii) *x*−1/2, −*y*+1/2, *z*+1/2.
